# Necroptotic cell death and immunomodulator release induced by T-cell engaging anti-lymphoma therapies

**DOI:** 10.1038/s41419-026-08993-7

**Published:** 2026-06-25

**Authors:** Demi Both, José Saura-Esteller, Anne Martens, Joanne Rietveld, Ingrid Derks, Morris Mes, Jan Willem Duitman, Anita Grootemaat, Nicole van der Wel, Arnon Kater, Marco Haselager, Eric Eldering

**Affiliations:** 1 Laboratory for Experimental Immunology, Amsterdam UMC location University of Amsterdam, Amsterdam, AZ The Netherlands; 2 Department of Hematology, Amsterdam University Medical Centers, University of Amsterdam, Amsterdam, BT The Netherlands; 3 Cancer Center Amsterdam, Cancer Immunology, Amsterdam, The Netherlands; 4 Amsterdam institute for Immunology and Infectious diseases, Amsterdam, The Netherlands; 5 Department of Medical Biology, Electron Microscopy Center Amsterdam, Amsterdam UMC, Amsterdam, The Netherlands; 6 Lymphoma and Myeloma Center Amsterdam, Amsterdam, BT The Netherlands

**Keywords:** Cancer immunotherapy, Cell death and immune response

## Abstract

Autologous cellular immunotherapies, which rely on cytotoxic T lymphocytes (CTLs), are increasingly applied in different B-cell malignancies. The general assumption is that CTLs exert their cytolytic function through granzymes that induce apoptosis in the target cell. However, the killing mechanism of immunotherapeutic CTLs is not clearly elucidated. Using T-cell redirecting bispecific antibodies (BsAbs) and chimeric antigen receptor (CAR) T-cells we assessed which cell death pathways were activated in B cell line models as well as in primary material from CLL patients. We demonstrate that for cytotoxic T-cell killing of malignant B-cells by any of the treatment strategies, caspase activity was not essential. We could also exclude a role for TNF or TRAIL-mediated pathways. Using electron microscopy, CAR T-cell and BsAb-mediated cell death showed a mixed apoptotic/necroptotic phenotype. This was corroborated by knockout and chemical inhibition of the essential necroptosis proteins RIPK1, RIPK3, and MLKL. Necroptotic death of target cells correlated with the release of HMGB1 in the supernatant as well as various immunomodulatory molecules from the T-cells. Besides known immune activators IFN-y, IL-17 and IL-6, also anti-inflammatory IL-10 and IL1-RA were released. Moreover, the quantity of these immunomodulators was differentially affected after application of apoptosis versus necroptosis inhibitors. Together, these data demonstrate that (CAR) T-cell-mediated killing of lymphoma cells has a necroptotic arm which is correlated with modulation of the wider immune response. They imply that manipulation of the apoptotic versus necroptotic balance in immunotherapy could affect engagement of the autologous immune response.

## Introduction

Over the past decade, significant progress has been made in the treatment of B-cell lymphomas and leukemias, however, therapeutic options for cases resistant to chemotherapy and targeted agents remain limited, representing a critical unmet clinical need [1, 2]. Autologous cell-based therapies, based on cytotoxic lymphocytes (CTLs), are increasingly implemented in treatment strategies of diffuse large B-cell lymphoma (DLBCL) and mantle cell lymphoma (MCL) and are actively investigated in the context of low-grade lymphomas [3]. Indeed, T-cell-redirecting bispecific antibodies (BsAbs), or chimeric antigen receptor (CAR) T-cells are part of the still expanding repertoire of CTL-based therapies.

Many lymphomas and leukemias resist both chemotherapy as well as targeted therapy, such as venetoclax, because of aberrant expression of anti- or pro-apoptotic proteins [4]. For instance, upregulation of the anti-apoptotic Bcl-2 protein is a hallmark of major subtypes of DLBCL, MCL, chronic lymphocytic leukemia (CLL) and follicular lymphoma. Upregulation of other anti-apoptotic proteins, such as Mcl-1 and Bcl-XL is also commonly found [4–9]. Moreover, structural and functional alterations in caspases, leading to apoptosis resistance, were observed in CLL, MCL and DLBCL respectively [9–11]. Even though leukemia and lymphoma patients can develop resistance to apoptotic stimuli, like chemotherapy or the Bcl-2 inhibitor venetoclax, these patients may still be salvaged by applying autologous T-cell therapy. In line with this, in vitro studies showed that venetoclax-resistant-cells are still sensitive to T-cell-mediated therapy [12]. This implies that CTL-mediated cell death occurs through either a different apoptotic route or that other forms of cell death are involved. Yet, the contribution of these alternative death pathways remains poorly defined in the context of modern immunotherapy.

Based on previous research, the consensus is that CTLs exert their cytolytic function mainly through either triggering of granule exocytosis or the Fas ligand pathway, both eventually leading to killing of the target cell through apoptosis [13–15]. Cell death induced by the granule exocytosis pathway mostly proceeds by intrinsic apoptosis [15, 16]. Granzyme B (GrB) either cleaves caspase-3 directly or it cleaves the BH3 protein Bid, resulting in Bax/Bak activation and mitochondrial outer membrane permeabilization (MOMP) [17–20]. On the other hand, Fas induction leads to extrinsic apoptosis by the DISC formation and caspase-8 activation, also leading to Bid cleavage [14, 21].

Recent research has uncovered additional forms of cell death that may contribute to CTL-mediated killing. This has gained attention since non-apoptotic forms of cell death appear more immunogenic than apoptosis. For instance, pyroptosis, which was originally described to be dependent on caspase-1 activity [22], was recently shown to be induced in cancer cells through granzyme A (GrA)-mediated cleavage of gasdermin B [23]. Additionally, necroptosis is predominantly activated by TNFR1 signaling and involves the necroptosome complex, including RIPK1, RIPK3 and MLKL, which is activated by a complex involving FADD in the absence of caspase-8 activity [24]. Other death receptors such as Fas or TRAIL-receptors can also induce necroptosis, which is known to be pro-inflammatory through release of so called ‘danger signals’. Nevertheless, anti-inflammatory effects have also been proposed [25]. In an experimental setting, induction of necroptosis by rapid dimerization via engineered variants of RIPK1 or RIPK3 in tumor targets resulted in enhanced immunogenicity, activation and clearance by CD8 T-cells [24, 26]. Vaccination with necroptotic cells led to prevention of tumor growth, pointing to the initiation tumor-directed immune activation by necroptosis [27]. These data imply that other forms of cell death might be evoked in tumor cells upon CTL-killing which may benefit immune-mediated tumor clearance.

We have previously observed that primary chronic lymphocytic leukemia (CLL) cells that become highly apoptosis-resistant after strong CD40 stimulation, can still be killed by CD3/CD19 bi-specific T-cell engagers [12]. This suggested contribution of a non-apoptotic component. Therefore, we here aimed to investigate in more detail the forms of cell death induced by different T-cell-based immunotherapies. We applied treatment by blinatumomab and CD19-directed-CAR T-cells in various types of B-cell lymphomas as well as CLL and found clear involvement of necroptosis. CTL-mediated killing led to the release of both pro- and anti-inflammatory cytokines from T-cells, production of which could be modulated upon treatment with caspase- or necroptosis inhibitors.

## Results

### Blinatumomab and CAR T-cell-mediated killing is not fully dependent on apoptosis via caspases and the Bax/Bak pathway

**Fig. 1:**
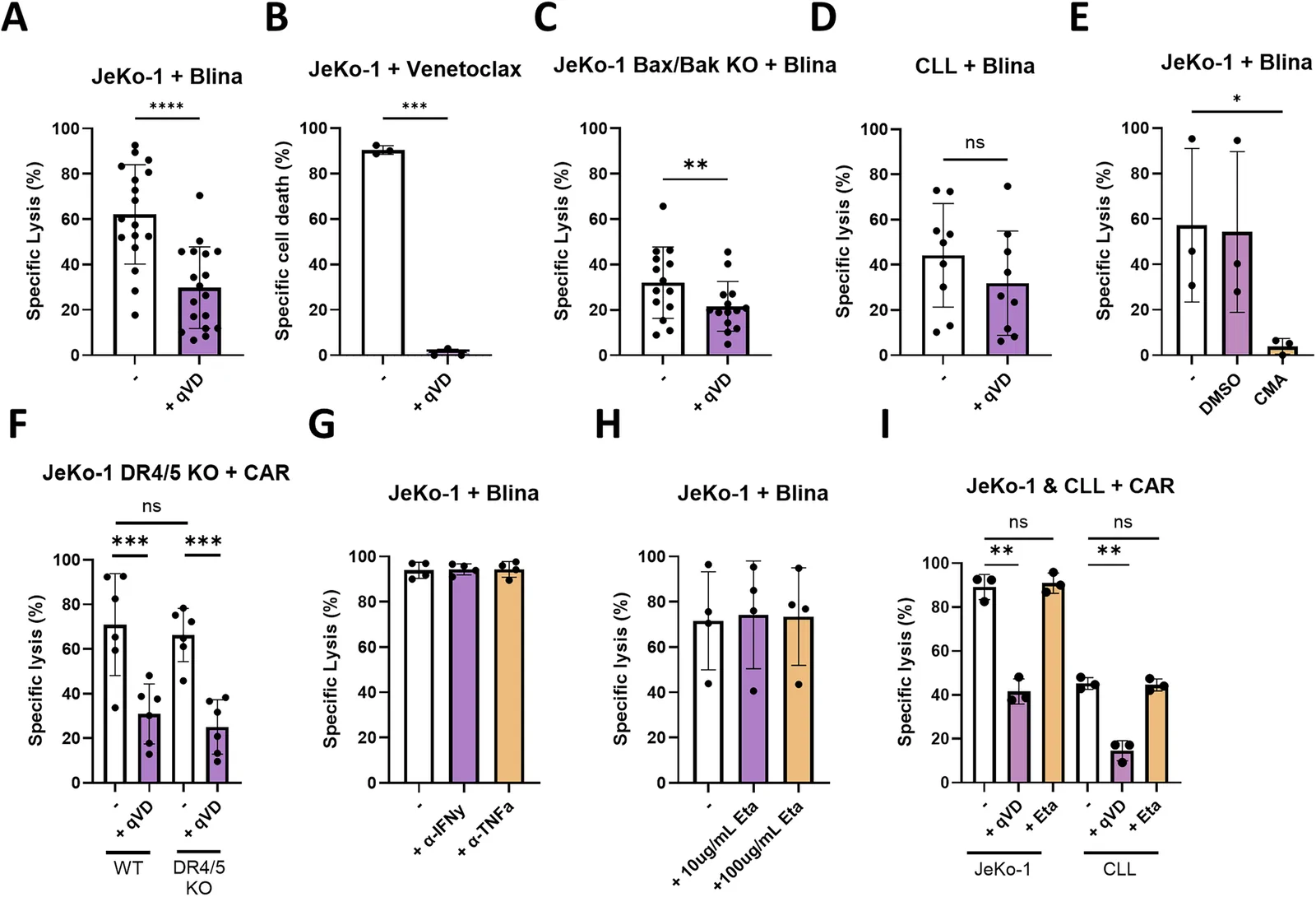
Determination of cell death pathways in CTL mediated killing of JeKo-1 and CLL cells. **A** Assessment of cytotoxicity of JeKo-1 after co-culture with T-cells in a 4:1 E:T ratio in the presence of 1 ng/mL blinatumomab with or without 20 µM qVD for 24 h (*N* = 18). **B** Cell death of JeKo-1 cells treated with 10 µM venetoclax in presence or absence of 20 µM qVD for 24 h (*N* = 3). **C** Assessment of cytotoxicity of JeKo-1 Bax/Bak KO cells after co-culture with T-cells in a 4:1 E:T ratio in the presence of 1 ng/mL blinatumomab with or without 20 µM qVD for 24 h (*N* = 14). **D** Assessment of cytotoxicity of CLL after co-culture with T-cells in a 4:1 E:T ratio in the presence of 10 ng/mL blinatumomab with or without 20 µM qVD for 48 h (*N* = 9). **E** Assessment of cell death of JeKo-1 cells which were co-cultured with 1 ng/mL blinatumomab and T-cells in a 4:1 E:T ratio that were pre-incubated with CMA or DMSO for 2 h prior to co-culture. (*N* = 3). **F** Assessment of cytotoxicity of JeKo-1 WT or DR4/DR5 KO cells after co-culture with CAR T-cells in a 4:1 E:T ratio with or without 20 µM qVD for 24 h (*N* = 6). **G** Cell death measured of JeKo-1 co-cultured with T-cells in a 4:1 E:T ratio and 1 ng/mL blinatumomab in the presence of 10 µg/mL anti-TNFα or anti-IFNγ (*N* = 4). **H** Cell death measured of JeKo-1 co-cultured with CAR T-cells in a 4:1 E:T ratio in the presence of 10 µg/mL Etanercept (*N* = 4). **I** Cell death measured of JeKo-1 and CLL co-cultured with CAR T-cells in a 4:1 E:T ratio in the presence or absence of 20 µM qVD or 10 µg/mL Etanercept (*N* = 3). Data are presented as mean ± SD and *P*-values were calculated using paired t tests or Friedman test (**E**). *P < 0.05, **P < 0.01; ***P < 0.001.

Using the MCL cell line JeKo-1 and primary CLL cells as targets, we tested usual cell death requirements for killing by healthy donor (HD) T-cells, triggered by the bispecific antibody blinatumomab (CD3xCD19 BsAb) or by anti-CD19 CAR T-cells. Blinatumomab triggered HD cells to kill JeKo-1 cells, which was only partially rescued by qVD (Fig. 1A). In comparison, cell death upon treatment with venetoclax (ABT-199), a strictly apoptotic stimulus, was fully inhibited by qVD (Fig. 1B). Additionally, blinatumomab still induced cell death in JeKo-1 cells that lack Bax/Bak [12, 28] and was only slightly reduced with qVD (Fig. 1C). When using primary CLL cells, the addition of qVD did not significantly reduce blinatumomab-induced cell death (Fig. 1D). To confirm dependence on granule exocytosis, T-cells were pre-treated with concanamycin A (CMA) [29]. which completely abrogated blinatumomab-mediated killing (Fig. 1E).

Next, we tested involvement of various cell surface death-inducing pathways after triggering with blinatumomab or CAR T-cells. First, JeKo-1 cells expressed almost no Fas and were not sensitive to Fas-induced cell death, as measured by treatment with Fas10 and Fas2 antibodies (Fig. S1A, B). Second, cell death by CAR T-cells in JeKo-1 DR4/5 double KO cells was not significantly different from killing of WT cells (Fig. 1F). Third, blocking IFNγ or TNFα did not reduce blinatumomab-mediated killing of JeKo-1 cells (Fig. 1G, H). CAR T-cells were also able to efficiently lyse JeKo-1 and CLL cells, and again caspase inhibition by qVD had only a partial effect (Fig. 1I). The addition of etanercept, which specifically blocks TNFR1 signaling, to Jeko-1 and CLL cells did not prevent CAR T-cell or blinatumomab-induced cell death (Fig. 1H, I). As a positive control, etanercept could fully block CXCL5 expression (Fig. S1C), which is dependent on TNFR1 signaling [30]. whereas the presence of GM-CSF, which is regulated by TNFR2 signaling [31]. was not affected. Expression of Gasdermin (GSDM) D and B was also relatively low in JeKo-1 cells (Fig. S1D). Combined, these results indicate that CTL-mediated cell death in these systems is partly apoptotic, strictly depends on granule exocytosis, and does not involve DR4/5, TNFα or IFNy.

### T-cell-mediated cell death displays non apoptotic cell fraction under caspase inhibition

**Fig. 2:**
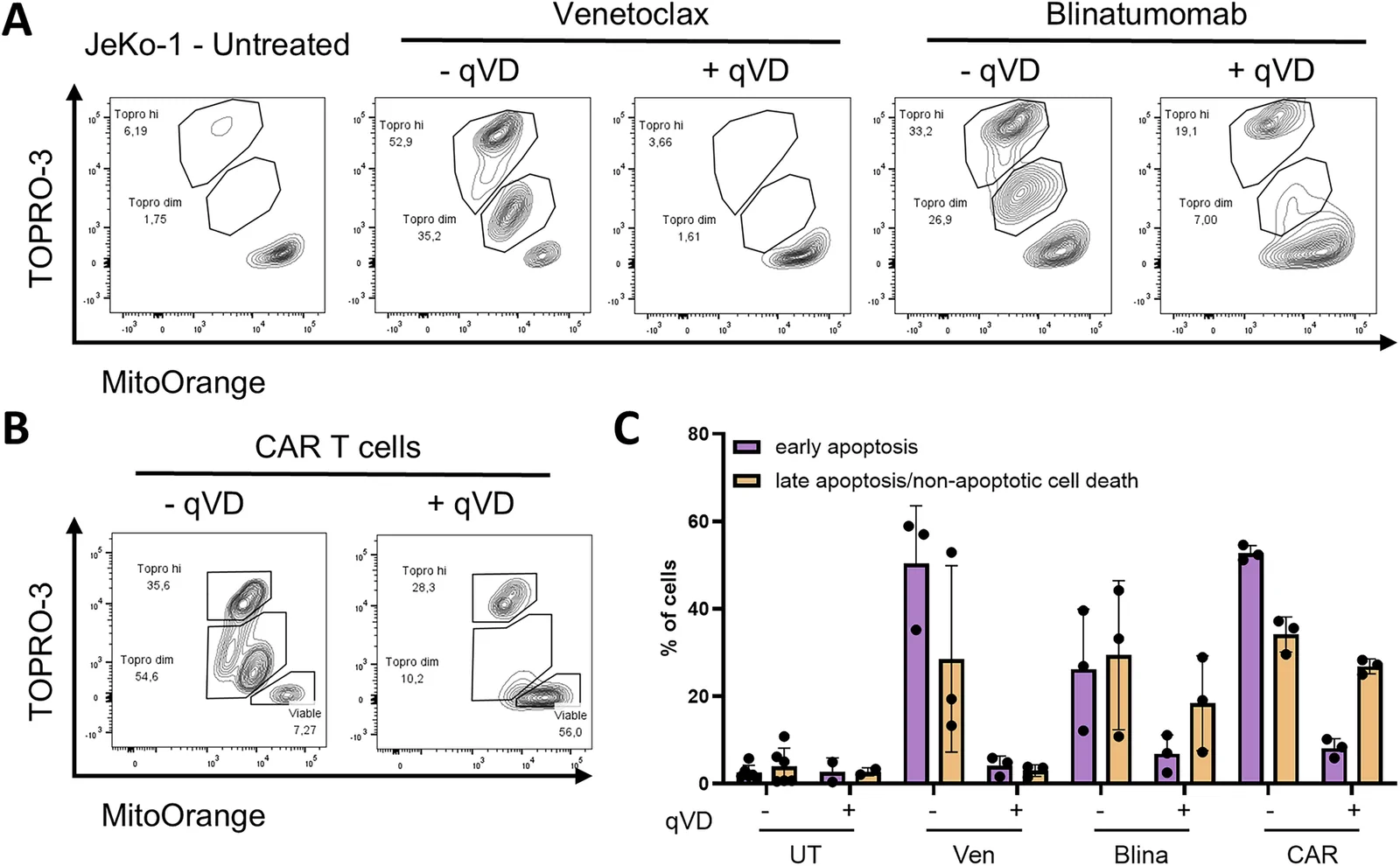
Identification of late apoptotic/non-apoptotic cell death populations of JeKo-1 cells in different treatment conditions. **A** Representative FACS plots of JeKo-1 stained with MitoTracker Orange and TOPRO-3 untreated or treated with 10 µM venetoclax or of 1 ng/mL blinatumomab in presence or absence of 20 µM qVD for 24 h (*N* = 3–6). **B** Representative FACS plots of JeKo-1 stained with MitoTracker Orange and TOPRO-3 after co-culture with CAR T-cells in a 4:1 E:T ratio in the presence or absence of 20 µM qVD for 24 h (*N* = 3). **C** Quantification of cell death populations of JeKo-1 (from **A**, **B**). Data are presented as mean ± SD.

Mitochondrial depolarization, and plasma membrane permeabilization are important components of intrinsic apoptosis [32]. Based on these characteristics, three distinct cell populations can be distinguished, namely viable (Mitotracker Orange^hi^TOPRO-3^low)^, early apoptotic (TOPRO-3^dim^) and late apoptotic/non-apoptotic (TOPRO-3^hi^). Apoptotic cell death by venetoclax resulted in these populations, first mitochondrial depolarization, followed by loss of membrane integrity (Fig. 2A). Under caspase inhibition, both populations disappear. Although identical populations were identified upon blinatumomab and CAR T-cell treatment (Fig. 2A, right panel and 2B), upon caspase inhibition the TOPRO-3^dim^ population and a fraction of the TOPRO-3^hi^ were lost. Similar data were obtained for CLL cells (Fig. S2). Quantification of the percentage of cells in the two stages of cell death is visualized in Fig. 2C. In conclusion, venetoclax induced classical apoptosis which was fully caspase dependent, whereas blinatumomab and CAR T-cell killing induced a second, caspase independent pathway.

### Electron microscopy reveals necroptotic morphology in blinatumomab-mediated cell death

**Fig. 3:**
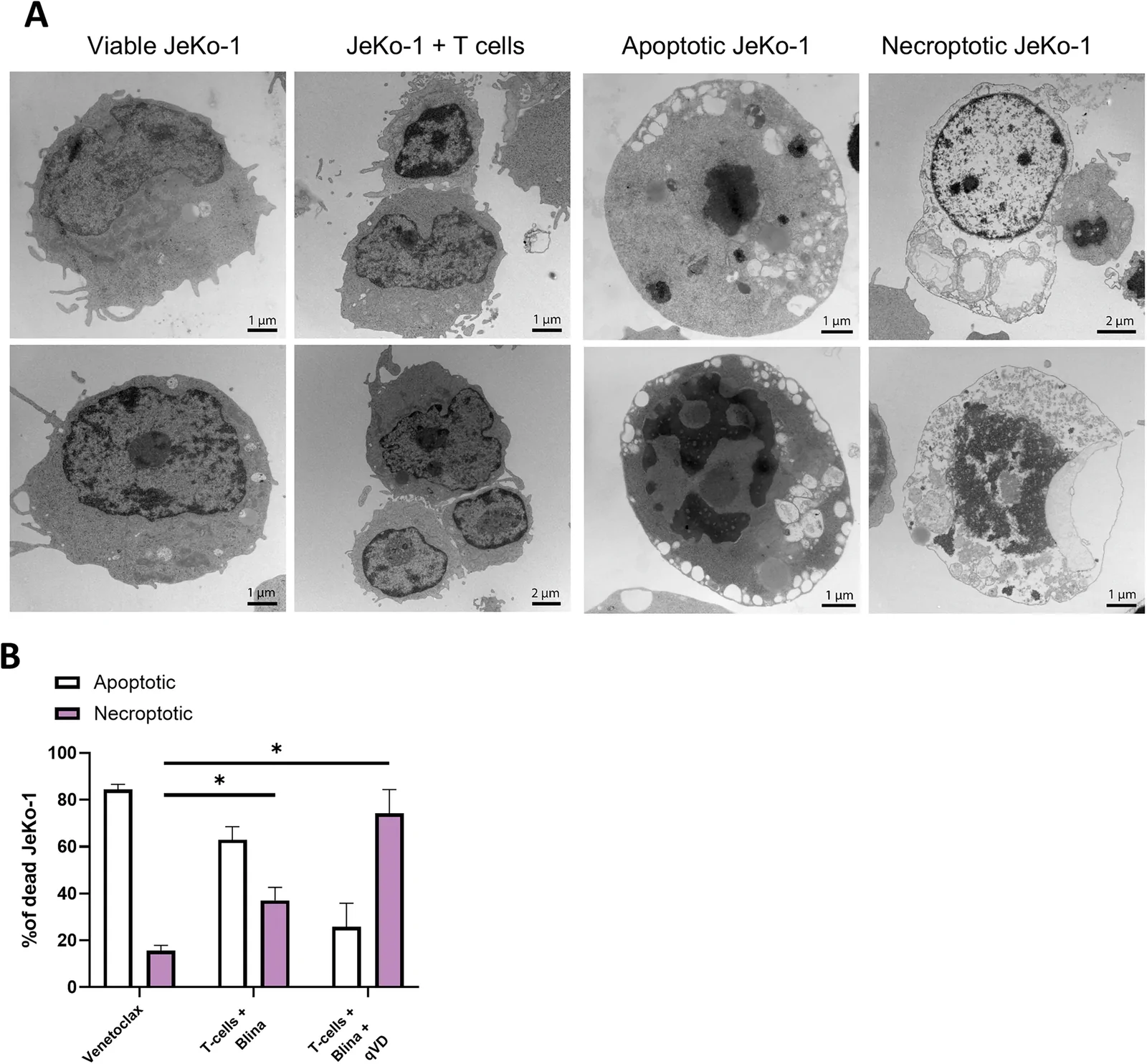
Electron microscopic analysis of cell death morphology in JeKo-1 cells in different treatment conditions. **A** Examples of electron microscopy images of living JeKo-1 cells, JeKo-1 cells with T-cells, apoptotic JeKo-1 cells and necroptotic JeKo-1 cells. Scale bars are indicated per panel. **B** Quantification of apoptosis and necroptosis after treatment of JeKo-1 cells with 10 µM venetoclax or after co-culture with T-cells in a 4:1 E:T ratio in presence of 1 ng/mL blinatumomab with or without 20 µM qVD for 24 h. Pictures were randomized and blinded before they were scored by four people. Shown are %apoptotic and %necroptotic JeKo-1 cells of dying JeKo-1 cells. Data are presented as mean ± SD and *P*-values were calculated using RM one-way ANOVA with Tukey’s multiple comparison test (**B**), *P < 0.05, **P < 0.01; ***P < 0.001.

Electron microscopy (EM) was performed to determine whether blinatumomab-mediated T-cell killing (in presence/absence of qVD) morphologically differed from apoptotic cell death. Representative examples of JeKo-1 cells with characteristic features of apoptotic versus necroptotic death are depicted in Fig. 3A. Apoptosis was characterized by mostly round cells, a clearly condensed nucleus, vacuole formation in the cytosol, electron dense cytosol and presence of organelles (Fig. 3A, third panel). Necroptotic cells have a clearly distinct morphology and were identified accordingly when they lost cytosol integrity, either highly condensed or dispersed nucleus with some condensed spots, and organelles were not visible or swollen and electron-lucent (Fig. 3A, fourth panel). Quantification showed that venetoclax-mediated cell death was predominantly apoptotic, whereas blinatumomab also caused a necroptotic morphology. This necroptotic population was already observed upon blinatumomab treatment without caspase inhibition and was further increased after addition of qVD (Fig. 3B). These results fit well with the flowcytometric analyses in Fig. 2 and combined they clearly support that blinatumomab and CAR T-cells induce apoptosis as well as necroptosis simultaneously.

### The RIPK1-RIPK3-MLKL-axis is involved in cell death induction by blinatumomab

**Fig. 4:**
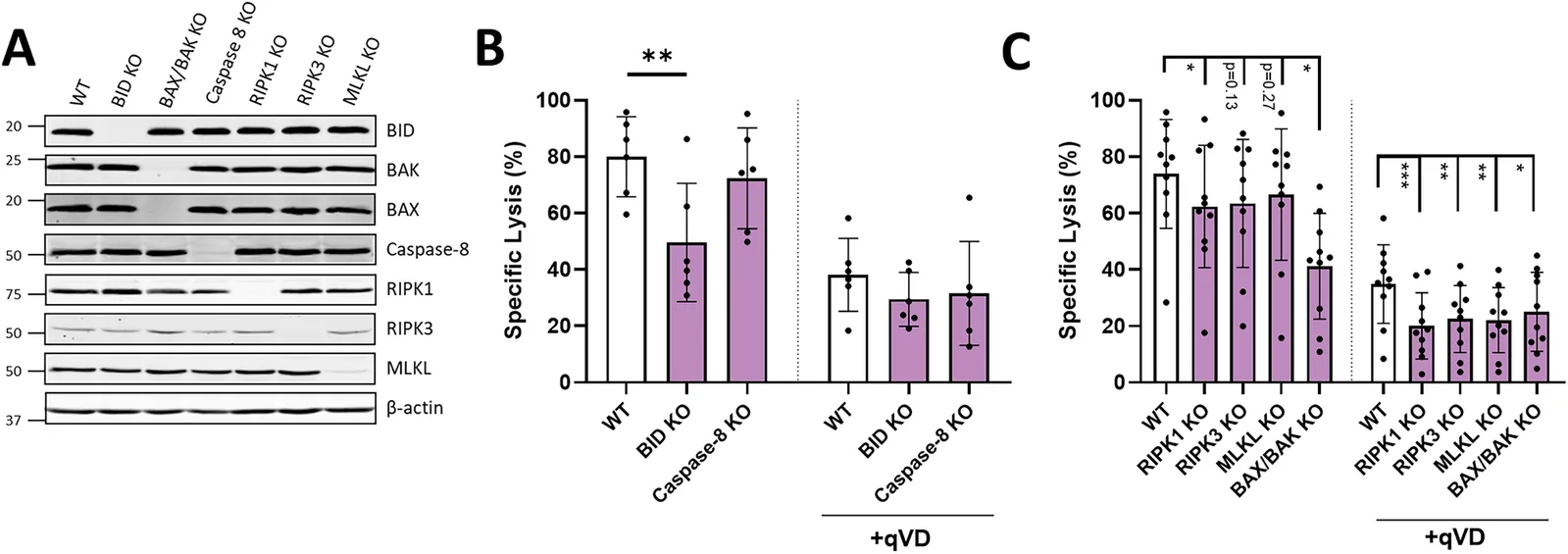
Effects of deleting distinct cell death proteins in JeKo-1 cells. **A** KOs for Bid, Bax and Bak, caspase-8, RIPK1, RIPK3 and MLKL in JeKo-1 cells were confirmed by western blot. **B** Specific lysis of WT JeKo-1 and Bid and caspase-8 KO cells after co-culture with T-cells in a 4:1 E:T ratio in the presence of 1 ng/mL blinatumomab with or without 20 µM qVD for 24 h (*N* = 5). **C** Specific lysis of WT JeKo-1 and RIPK1, RIPK3, MLKL and Bax/Bak KO cells after co-culture with T-cells in a 4:1 E:T ratio in the presence of 1 ng/mL blinatumomab with or without 20 µM qVD for 24 h (*N* = 10). Data are presented as mean ± SD and *P* values were calculated using RM one-way ANOVA followed by Dunnet posthoc tests, *P < 0.05, **P < 0.01.

To determine the contribution of key necroptotic and apoptotic regulators, gene KOs were made by CRISPR technology in JeKo-1 cells (Fig. 4A). As expected, based on current knowledge, Bax/Bak double KO blocked intrinsic apoptosis by venetoclax, Bid, Bax/Bak or Caspase-8 KO prevented extrinsic apoptosis by SuperKillerTrail (SKT), and RIPK1, RIPK3 and MLKL KOs prevented necroptotic death by SKT+qVD (Fig. S3A). Interestingly, also caspase-8 KO rescued cell death induced by SKT+qVD, showing its essential role in the complex formation of both apoptosis and necroptosis [33]. Apoptosis-related proteins Bid and Bax/Bak were involved in blinatumomab-mediated killing (Fig. 4B), but caspase-8 was not, in accordance with the expected direct cleavage of Bid by GrB, thereby activating the intrinsic apoptosis pathway [18]. RIPK1 KO affected blinatumomab-mediated T-cell killing without caspase inhibition. Genetic perturbation of RIPK3 and MLKL showed effects that were comparable in magnitude to RIPK1 perturbation. Upon addition of qVD, absence of RIPK1, RIPK3 and MLKL further prevented cell death significantly (Fig. 4C). Blinatumomab treatment slightly increased phosphorylation of RIPK1 on S161 [34, 35], which was correlated with the induction of cell death (Fig. S3B, C). Although overall cell death was reduced by the addition of qVD, RIPK1 phosphorylation was further increased, which suggests activation of the pathway. This effect was comparable to the positive control for necroptosis induction using a combination of SKT, qVD plus SMAC mimetic. These observations suggest a necroptotic component in blinatumomab-mediated lysis which is dependent on RIPK1, RIPK3 and MLKL signaling. Yet, the fraction of cell death that remains in these KO cells, suggests this model might not be optimal, probably because of the scaffold function of the RIPK proteins or their ability to induce necroptosis independently of each other.

### BsAb and CAR T-cell-mediated cell death can be inhibited by combining apoptosis and necroptosis inhibitors

**Fig. 5:**
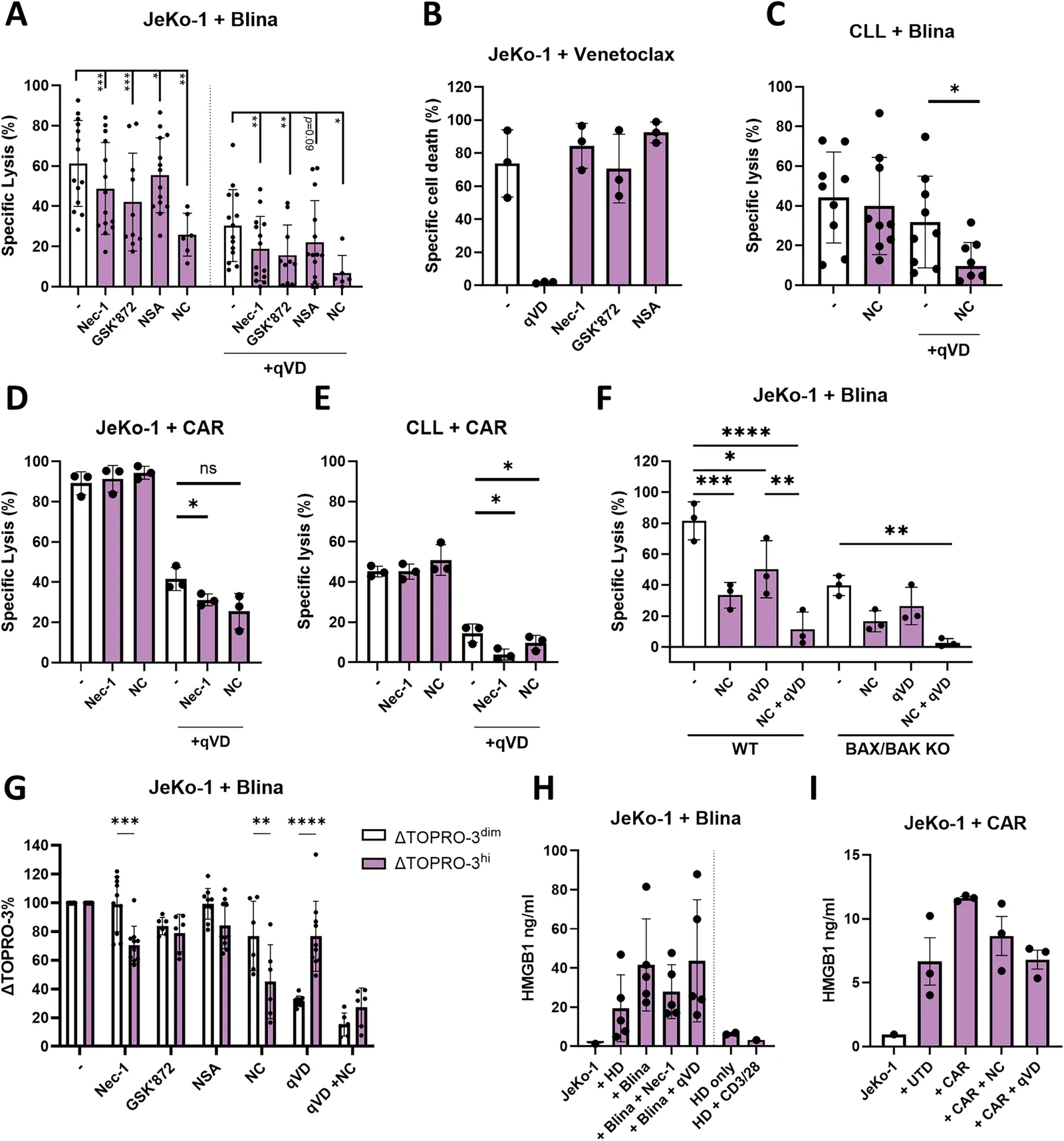
CTL mediated killing in presence of qVD in combination with necroptosis inhibitors. **A** Specific lysis of JeKo-1 cells after co-culture with T-cells in a T-cells in a 4:1 E:T ratio in the presence of 1 ng/mL blinatumomab with or without 20 µM qVD, 50 µM Nec-1, 0.63 µM GSK’872, 1.25 µM NSA or necroptosis cocktail (NC; 50 µM Nec-1, 2.5 µM GSK’872 or 2.5 µM NSA) for 24 h (*N* = 10–15). **B** Specific cell death of JeKo-1 cells treated with 10 µM venetoclax with or without 20 µM qVD, 50 µM Nec-1, 0.63 µM GSK’872 or 1.25 µM NSA for 24 h (*N* = 3). **C** Specific lysis of CLL cells after co-culture with T-cells in a 4:1 E:T ratio in the presence of 10 ng/mL blinatumomab with or without 20 µM qVD and NC (50 µM Nec-1, 2.5 µM GSK’872 or 2.5 µM NSA) for 48 h (*N* = 9). Specific lysis of JeKo-1 (**D**) and CLL (**E**) cells after co-culture with CAR T-cells in a 4:1 E:T ratio with or without 20 µM qVD, 50 µM Nec-1 or NC (50 µM Nec-1, 0.63 µM GSK’872 or 1.25 µM NSA) for 24 h (*N* = 3). **F** Specific lysis of JeKo-1 WT and BAX/BAK KO cells after co-culture with T-cells in a T-cells in a 4:1 E:T ratio in the presence of 1 ng/mL blinatumomab with or without 20 µM qVD and necroptosis cocktail (NC; 50 µM Nec-1, 2.5 µM GSK’872 or 2.5 µM NSA) for 24 h (*N* = 3). **G** Frequency of TOPRO-3 high and dim populations in JeKo-1 cells relative to blinatumomab (-) treatment (N = 4-10). **H**, **I** HMGB1 levels from co-cultures of JeKo-1 with T-cells in a 4:1 E:T ratio in the presence of 1 ng/mL blinatumomab (*N* = 6) or CAR T-cells (*N* = 3) with and without 20 µM qVD or 50 µM Nec-1 for 24 h measured with ELISA. Data are presented as mean ± SD and *P*-values were calculated using mixed effects model for ANOVA followed by Dunnet posthoc (**A**), paired t-test (**C**), RM one-way ANOVA followed by Dunnet posthoc (**D**, **E**), or two-way ANOVA followed by Šidák’s multiple comparison (**F**, **G**), *P < 0.05, **P < 0.01.

We next investigated inhibition of RIPK1/3 kinase activity and the oligomerization of MLKL using specific inhibitors [36–38]. Since necroptosis is mostly detectable in the presence of caspase inhibition [39], combinations of inhibitors of RIPK1 (Nec-1), RIPK3 (GSK’872) and MLKL (NSA) with and without qVD were tested on JeKo-1 cells (Fig. 5A). Blinatumomab treatment of JeKo-1 cells induced cell death, which was slightly though significantly reduced by all three inhibitors as well as a necroptosis cocktail (NC) containing all three inhibitors, already without qVD. When combined with qVD, the remaining blinatumomab-induced cell death was further reduced by inhibition of Nec-1, GSK’872 and the NC. In contrast, induction of cell death by venetoclax was not affected by any of the necroptosis inhibitors (Fig. 5B). Similar results were observed in CLL cells treated with HD cells and blinatumomab, where the NC significantly reduced cell death when combined with qVD (Fig. 5C). Combination of necroptosis inhibitors with qVD was also able to inhibit cell death in response to CAR T-cells generated from three independent donors in both JeKo-1 (Fig. 5D) and CLL (Fig. 5E) cells, compared with the effect of qVD alone. CTL-mediated lysis could be inhibited by the addition of Nec-1 or the NC to qVD. Killing of JeKo-1 cells was not fully rescued using inhibitors, likely due to high efficiency of CAR T-cell-mediated killing. In CLL cells, where the cytotoxic activity was slightly less pronounced, cell lysis was completely abrogated by Nec-1 and qVD. The combination of qVD and NC on Bax/Bak KO cells fully inhibited blinatumomab-induced lysis (Fig. 5F). Moreover, when looking into TOPRO-3^hi^ and TOPRO-3^dim^ populations the addition of Nec-1 and the NC showed a significant reduction of the TOPRO-3^hi^ population, and RIPK3 and MLKL followed a similar trend (Fig. 5G), suggesting cell death in this non-apoptotic population is dependent on these proteins. Necroptotic cells secrete the damage-associated molecular pattern (DAMP) molecule HMGB1 [40]. In accordance, HMGB1 levels in supernatant were increased upon triggering of cell death of JeKo-1 cells by blinatumomab and CAR T-cells and was reduced in the presence of RIPK1 inhibition by Nec-1 (Fig. 5H, I). In comparison, JeKo-1 cells treated with venetoclax did not secrete HMGB1 and HMGB1 secretion following necroptosis induction by combination treatment of SKT, BV-6 and qVD was strongly induced and completely abolished by Nec-1 (data not shown). Combined, these results further support that T-cell-mediated killing of malignant B-cells contains a necroptotic component, which is dependent on combined signaling of RIPK1, RIPK3 and MLKL.

### T-cell-mediated cell death induces release of immunomodulatory molecules

**Fig. 6:**
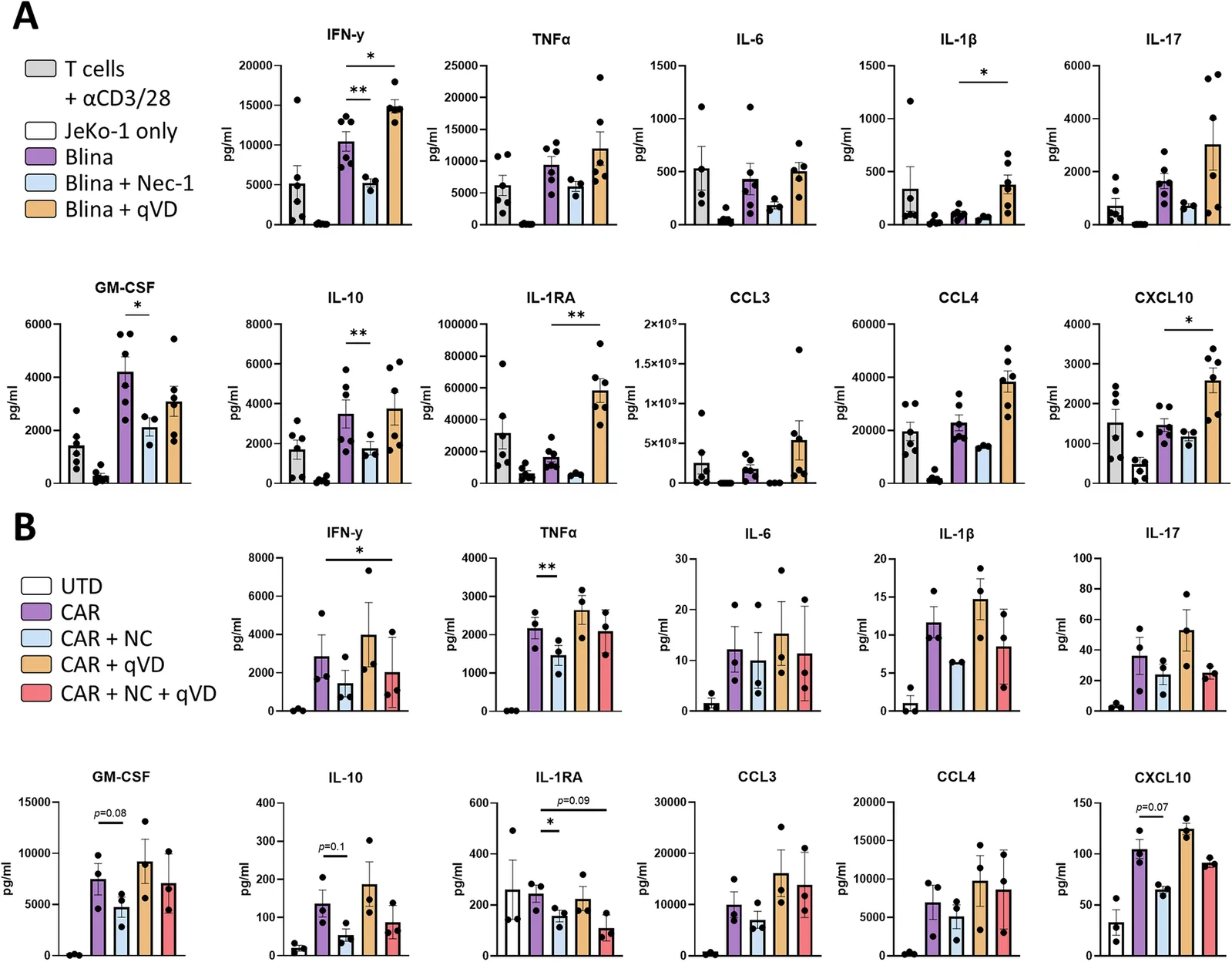
Analysis of culture supernatants of killing JeKo-1 by CTLs. **A** Cytokine and chemokine levels measured in supernatant from JeKo-1 cells only and co-cultures of JeKo-1 with T-cells in a 4:1 E:T ratio in the presence of anti-CD3/CD28 activated T-cells (positive control), 1 ng/mL blinatumomab, 20 µM qVD or 50 µM Nec-1 for 24 h (*N* = 3–6) measured with Luminex. **B** Cytokine and chemokine levels measured in supernatant from co-cultures of JeKo-1 with untransduced (UTD) or CAR T-cells in a 4:1 E:T ratio in the presence or absence of 20 µM qVD, necroptosis cocktail (NC; 50 µM Nec-1, 0.63 µM GSK’872 and 1.25 µM NSA) or the combination of qVD and NC for 24 h (*N* = 3) for 24 h measured with Luminex. Data are presented as mean ± SD and *P*-values were calculated using mixed effects model for ANOVA followed by Dunnet posthoc (**A**) or RM one-way ANOVA followed by Dunnet posthoc (**B**), *P < 0.05, **P < 0.01.

Since CTL-mediated killing induces HMGB1 release, we examined secretion of immunomodulatory molecules. Supernatants of both JeKo-1 and CLL cells killed by T-cells with blinatumomab or CAR T-cells were analyzed by multiplex assay for cytokines and chemokines. This revealed that in comparison to standard anti-CD3/CD28 T-cell stimulation, levels of several immunomodulatory molecules such as IFN-γ, TNFα, IL-6, IL-1β, IL-17, GM-CSF, IL-10, IL-1RA, CCL3, CCL4 and CXCL10 all increased upon blinatumomab-mediated killing (Fig. 6A, B). Upon addition of qVD or Nec-1 to blinatumomab treatment, most cytokines showed a pattern of elevated levels after caspase inhibition and a reduction by the necroptosis inhibitor. Exception to this trend were observed for GM-CSF, where qVD did not increase the concentration in the supernatant, but levels were still reduced by addition of Nec-1.

Comparable patterns of cytokines and chemokines were observed in supernatants derived from co-cultures of JeKo-1 with CAR T-cells (Fig. 6B). In comparison with blinatumomab, GM-CSF aligned with the overall trend, whereas IL-1RA did not. In this setting we also added the combination of qVD and NC to the co-culture which resulted in intermediate cytokine release. Moreover, the same effects were observed in CLL samples treated with CAR T-cells, although concentrations were lower compared to JeKo-1 (Fig. S4).

Using an engineered system to rapidly dimerize RIPK1 or -3, it has been reported that necroptotic cells actively transcribe and secrete inflammatory cytokines such as IL-6 which can enhance immune-mediated clearance of cancer cells [24, 26]. However, intracellular staining for IL-6 during blinatumomab-triggered CTL attack showed that the CD4^+^ T-cell population was responsible for production, while the JeKo-1 targets and also CD8 T-cells hardly produced this cytokine (Fig. S5).

These results show that upon killing by CTLs a spectrum of both pro- and anti-immunogenic compounds are released from T-cells, and the levels are affected by the predominant mode of killing of target cells. The overall findings suggest a potential role for RIPK1-RIPK3-MLKL necroptosis in immunomodulation.

## Discussion

Application of BsAbs and CAR T-cells is an emerging clinical strategy in B-cell malignancies. As main finding, we show that blinatumomab and anti-CD19 CAR T-cells kill JeKo-1 and primary CLL cells through combined apoptotic and necroptotic mechanisms. It has been reported that both pyroptosis and necroptosis can be induced by granules from CTLs [23, 24, 41]. Our data points into the direction of the latter, based on morphological (EM) criteria that point towards necroptosis and the fact that inhibitors for RIPK1, RIPK3 and MLKL were able to strongly reduce cell death.

Based on our data we favor a model where apoptosis and necroptosis can be induced in parallel. Although the largest proportion of the observed CTL-mediated cell death was apoptotic, clear signs of necroptosis were observed also in absence of caspase inhibition. This is based on morphological data as well as cytokine release. Application of caspase- versus necroptosis inhibitors pushed cell death towards the alternative pathway, with effects on release of inflammatory cytokines and immunomodulators. However, we do not know whether occurrence of the two cell death pathways can be in one cell or in separate cells. Since we did not pinpoint a trigger for necroptosis, we cannot fully exclude that the two cell death pathways occur hierarchically, with apoptosis occurring first.

The addition of CMA fully abrogated blinatumomab-induced cytotoxicity, underscoring the critical role of granule exocytosis. GrA can trigger pyroptosis by cleavage of GSDMB [21, 23]. Since both GSDMB and D expressions were hardly detectable in JeKo-1 cells, the contribution of this route seems unlikely. GrB is well-known to cleave substrates in the pathway of caspase dependent apoptosis [15, 16, 20, 42] while necroptosis mediators RIPK1 and RIPK3 are activated by (auto-)phosphorylation, although the detailed mechanisms or triggers of their activation are not fully elucidated [34, 35, 43, 44]. Whereas previous studies describe DR or TNFR signaling pathways as essential for killing by T-cells or NK-cells [45, 46]. We show that necroptosis in the killing models applied here was not dependent on either of these. In addition, there appeared to be no role for TNF or IFN-γ in CTL killing induced by BsAbs or CAR T-cells as opposed to previous reports [46–48]. A potential explanation could be that the T-cells engaged via CARs or using blinatumomab are not engaged via an immunological synapse involving classical MHC-TCR interaction. Therefore, the distinct spatial organization of the synapse may not result in engagement of downstream NF-κB, MAPK, or NFAT that upregulate TNF gene expression [46, 49]. Additionally, the role of IFN-γ was emphasized mostly in murine models or human CD4 CAR T-cells, which are not directly comparable to our model using short-term killing in cell cultures [48, 50].

CRISPR-mediated knockout of RIPK1 or RIPK3 did not completely prevent CTL-mediated killing, in contrast to the effects observed with the combination of chemical inhibitors of these proteins. Since Nec-1 was shown to prevent RIPK3 phosphorylation by inhibition of RIPK1 and thereby preventing necroptosis, RIPK1 was believed to be essential for induction of necroptosis [51, 52]. However, subsequent studies revealed that necroptosis can still take place in the absence of RIPK1, where triggering by TLRs or TNF, interferons led to RIPK3-dependent necroptosis, likely due to RIPK3 oligomerization [53–55]. Therefore, RIPK1 might rather function as a scaffold and necroptosis is triggered by autophosphorylation of RIPK3. Co-immunoprecipitation experiments showed that chemical inhibition of RIPK1 by Nec-1 disrupts association of MLKL and RIPK3, thereby inhibiting necroptosis, which was dependent on RIPK1 presence [53]. In line with this, in vitro studies RIPK1 failed to phosphorylate RIPK3 [56]. Together this indicates that presence of RIPK1 is indeed not always necessary to induce necroptosis. Whether similar discrepancies also occur in RIPK3 KO versus chemical inhibition is not assessed to our knowledge.

A topic for further research is identifying the trigger of necroptosis. A speculative stimulus could be the activation of ZBP-1, which may induce RIPK3 and MLKL, causing lysis, possibly via GrA-mediated mitochondrial DNA release [57–59]. However, since RIPK1 inhibition reduced cell death, this pathway alone seems unlikely.

Activation of RIPK1 could be determined by phosphorylation of S161 and S166, both present in the activation loop of the protein [34, 35]. We aimed to measure this phosphorylation following blinatumomab-mediated killing but observed only a minor increase, although the percentage increased upon blinatumomab with qVD even though overall cell death decreased. Since treatment with the potent necroptosis inducing combination of SKT, BV6 and qVD did also not increase the percentage of pRIPK1 positive cells to a high extent, this could point at technical limitations rather than a physiological effect and could be subject for further research.

Necroptosis is typically characterized as a pro-inflammatory form of cell death, owing to the release of DAMPs and pro-inflammatory cytokines and chemokines [26, 27, 40]. From IL-17 it is known that it is produced by Th17 CD4^+^ T-cells [60]. But also IL-6 can be produced by (activated) CD4^+^ T-cells [61]. Interestingly, we also observed an increased release of anti-inflammatory cytokines, which are likely produced by Tregs[62]. Overall, the cytokine release followed a consistent pattern: levels were elevated upon treatment with either blinatumomab or CAR T-cells, further enhanced by caspase inhibition, and reduced upon RIPK1 inhibition. Whether the concurrent release of both pro- and anti-inflammatory mediators serves to modulate excessive inflammation or potentially counteracts the beneficial effects of necroptosis on lymphoma clearance requires further investigation. Intracellular cytokine staining revealed that the anti-inflammatory cytokine IL-10 was produced at later time points by CD4⁺ T-cells (data not shown), suggesting that regulatory T-cells (Tregs) are likely the primary source of this cytokine [63]. Additionally, it remains to be determined whether their expression is dependent on RIPK1 and NF-κB signaling [26].

Clinical trial data in CLL shows that venetoclax resistant patients can still be targeted using CAR T-cells, in which similar efficacy is observed as in non-resistant patients, also highlighting a possible role of non-apoptotic cell death in vivo [64]. Clinically pushing cells into the direction of more immunogenic pathways could benefit CTL-mediated therapies. Therefore, combination therapy of necroptosis-inducing compounds with BsAbs or CAR T-cells could be promising and subject for future research. Such mechanisms are already described, for instance by SMAC mimetics such as Birinapant [65–68].

In conclusion, we show that T-cell-mediated killing of lymphoma cells by CARs or blinatumomab is partly necroptotic, which is enhanced upon caspase inhibition and leads to the release of immunomodulatory molecules. Since necroptosis leads to enhanced tumor clearance, boosting the necroptotic component of CTL-mediated killing using BsAbs or CAR T-cell therapy in lymphoma may enhance the efficacy of these treatment strategies.

## Materials and methods

### Cell lines and primary cells

Peripheral blood mononuclear cells (PBMCs) were isolated from buffy coats from Sanquin Blood Supply (Amsterdam, The Netherlands) using Ficoll-Plaque (VWR). All samples were cryopreserved in the vapor phase of liquid nitrogen. PBMCs, JeKo-1 and Ramos FSA cells (obtained from DSMZ) were cultured in RPMI 1640 medium (Thermo Fisher Scientific). Cell lines were authenticated by short tandem repeat analysis and the absence of mycoplasma was routinely checked. All medium was supplemented with 10% fetal calf serum (FCS) and 1% penicillin/streptomycin.

### Generation of knockout cell lines

Knockout (KO) JeKo-1 cells were generated using CRISPR/Cas9 technology. JeKo-1 Bax/Bak and DR4/DR5 KOs were generated as described before [12, 28]. The Bid and Caspase 8 KOs were generated in similar fashion. To generate RIPK1 and RIPK3 KO cells three sgRNAs per gene were cloned in the LentiGuide-puro plasmid, a gift from Feng Zhang (Addgene #52963). JeKo-1 cells were first transduced with Lenti-Cas9-2A-Blast, a gift from Jason Moffat (Addgene #73310). After selection with blasticidin (Sigma), the Cas9-expressing cells were transduced with a pool of three gRNAs per gene. gRNA sequences were described before and are listed in Supplementary Table 2 [69–71]. After transduction with the gRNAs the cells were selected with puromycin (Sigma), single cell cloning was performed and KOs confirmed by SDS-PAGE.

### Production of CAR lentivirus and CAR T-cell transduction

Retroviral supernatant was produced with transient transfection of Phoenix-Ampho (ATCC) cells with a plasmid containing hCD19 CAR and separate plasmids encoding gag-pol (pHIT60), and envelop (pCOLT-GALV) (Roche) following a standard Calcium Phosphate protocol [72]. Supernatant was harvested at 24 h and 48 h. To remove cellular debris, the supernatant was collected and centrifuged for 5 min at 1500 rpm and concentrated using RetroX concentrator (Takara Bio). A 293vec-RD114 stable virus producer cell line was produced expressing the human CD19 CAR [73]. 293vec-RD114 cells (0.3 × 10^6^ per well) were plated in a gelatin-coated 6-well plate one day before transduction. To transduce the cells, 1 ml of medium was removed and 1 ml of concentrated (Phoenix-Ampho-derived) γ-retroviral CD19 CAR was added together with 4ul/ml polybrene. The cells were spinoculated for 1 h at 3000 rpm at room temperature and were incubated for 24 h at 37 °C. Thereafter, the transduced CD19 CAR 293vec-RD114 were expended. At ~90% confluence, the viral vector containing supernatants was harvested, spun down at 1500 rpm to remove any cells or debris and concentrated using Retro-X concentrator (TakaraBio). For CAR T-cell transduction, T-cells were isolated using EasySep Human T-cell Enrichment Kit (Stemcell Technologies) and activated using anti-CD3/CD28 Dynabeads (Thermo Fisher) in a 3:1 bead:T-cell ratio in RPMI supplemented with 10% FCS, 1% penicillin/streptomycin. Two hits of T-cell transduction were performed on subsequent days with freshly harvested CD19 CAR 293vec-RD114 virus, starting on day two after T-cell activation. First, the virus supernatant containing RetroX was centrifuged 45 min 4 °C and resuspended in complete RPMI medium to concentrate the virus. 1 ml of medium was removed from the T-cells, 1 ml of concentrated virus was added to the T-cells, together with 4 ug/mL polybrene, and plates were spinoculated for 1 h at 3000 rpm. After 6 h, the medium was replaced with complete culture medium supplemented with 50 IE/mL rhIL-2 (Proleukin®; Novartis). After 72 h, the transduction efficiency was determined using biotinylated Protein L (Thermo Scientific) and PE-Streptavidin (BD Biosciences) staining by flow cytometry. For each donor an untransduced (UTD) T-cell fraction was used as negative control.

### Electron microscopy

JeKo-1 and T-cell co-cultures were fixed 1:1 with 0.2 M PHEM (240 mM piperazine-N,N′-bis(2-ethanesulfonic acid) (PIPES), 100 mM HEPES, 8 mM MgCl_2_, and 40 mM EGTA), 4% paraformaldehyde (Merck), and 0.4% glutaraldehyde (Merck) fixative. After washing with phosphate buffer the specimens were post-fixed with a solution of 1% OsO_4_ in water. Subsequently, the specimens were dehydrated in an ethanol series and embedded in epoxy resin (LX112). For electron microscopic analysis ultrathin (70 nm) sections of the samples were cut with a diamond (Diatome) knife on a Leica Ultracut UC6 ultramicrotome and collected on formvar-coated grids. The sections were counterstained with uranyl acetate (EMS) and lead citrate (Laurylab). All samples were examined and photographed in a FEI Tecnai T12 transmission electron microscope at the Electron Microscopy Centre Amsterdam. In total, 62 pictures showing multiple cells were randomized and blinded, and subsequently scored independently by four people. For quantification percentages of apoptotic and necroptotic cells of more than 150 cells per condition were calculated from the dead JeKo-1 population.

### Flow cytometry

Viability of the target cells was assessed using TO-PRO-3 (Invitrogen) and MitoTracker Orange (Invitrogen) or DioC6 (Thermo Fisher) using flow cytometry. For membrane marker expression cells were washed with PBA (PBS, 0.5% BSA and 0.02% Sodium Azide) and stained using titrated fluorescently labeled antibodies for 20 min on ice. For intracellular staining of cytokines, cells were pre-treated with Brefeldin A/Monensin mix for 4 h prior to staining. Antibodies are listed in Supplementary Table 3. Fixable Viability Dye eFluor™ 780 (eBioscience) was used as viability marker. After antibody staining, samples were washed and fixed (eBioscience kit #5223) using PBA and acquired on BD FACS Canto or Fortessa flow cytometers and analysed with FlowJo v10. For the pRIPK1 intracellular staining cells were collected and stained with the Fixable Viability Dye eFluor™ 780 for 20 min at room temperature. Cells were then fixed with BD Cytofix™ (BD Biosciences) for 15 min at room temperature and permeabilized for 10 min with BD Phosphoflow™ (BD Biosciences) on ice. Finally, cells were incubated for 1 h at room temperature with 2.5 μg/mL pRIPK1 (ser161) antibody (Proteintech) diluted in PBA and washed 5 times with PBA prior to acquisition. Wash steps with PBS were performed between each incubation to ensure removal of previous reagents.

### Cytotoxicity assay

Cell lines were labeled with cell trace violet (CTV) (Thermo Fisher Scientific) according to manufacturer’s instructions and co-cultured with healthy donor PBMCs or CAR T-cells in a 4:1 effector to target (E:T) ratio. For JeKo-1 cells 1 ng/mL and CLL cells 10 ng/mL blinatumomab (Amgen) was added during co-cultures to assess bispecific antibody mediated killing. Where indicated, T-cells were pretreated for 2 h with 1 µM concanamycin A (CMA) or dimethyl sulfoxide (DMSO) as a control. Treated T-cells were thoroughly washed before using in the co-culture. Inhibitor concentrations were titrated for optimal results. Where indicated, 20 µM of the pan-caspase inhibitor QVD-OPh (qVD, APExBIO), 50 µM necrostatin-1 (Nec-1) 0.16 µM or 0.63 µM GSK’872, 1.25 µM necrosulfonamide (NSA) 10 µg/mL anti-TNFα or anti-IFNγ or 10 or 100 µg/ml etanercept were added to JeKo-1 cocultures. For CLL a necroptosis cocktail (NC) containing 50 µM Nec-1, 2.5 µM GSK’872 and 2.5 µM NSA, was used. To induce apoptosis, JeKo-1 cells were treated with 10 μM venetoclax (ABT-199) or 25 ng/mL SuperKillerTrail (SKT) for 24 h. Necroptotic cell death was induced in JeKo-1 using 25 ng/mL SKT, 20 µM qVD and 5 μM BV-6 (when indicated) for 24 h. Controls for T-cell activation consisted of PBMC stimulation for 24 h with CD3 (91 ng/mL, clone 1XE) and CD28 (3 µg/mL, clone 15E8) antibodies. Specific lysis of target cells was calculated as (% target cell death in treated sample - % cell death target cells in medium control)/(100 – % cell death target cells in medium control) * 100%. Samples were excluded when cell death in medium controls exceeded 50%. Supernatants of these co-cultures were saved at -20 °C for further analysis.

### Analysis of cytokines and chemokines in supernatants by Luminex

HMGB1 release was measured in supernatant from cytotoxicity assays using a HMGB1 ELISA kit (Tecan) according to manufacturer’s instructions. Cytokines, chemokines and other analytes in frozen supernatant from cytotoxicity assays were measured using the human XL cytokine magnetic Luminex performance assay (#LKTM014) and an 18-plex custom human Luminex discovery assay (#LXSAHM, R&D systems) using a LX-200 instrument system (R&D systems) according to standard protocol. Data quality was evaluated based on the bead count, which represents the number of replicates measured for each biomarker concentration. Measurements with fewer than 25 beads were considered low quality and excluded from analysis. Values falling below the lowest point of the calibration curve were replaced with 0.5 times the minimum detectable value, usually corresponding to a biologically negligible concentration. When possible, samples exceeding the highest calibration point were extrapolated. Absolute cytokine concentrations are displayed.

### Statistical analysis

Data was checked for normality by a Shapiro-Wilk test. Treatment conditions were compared to the untreated condition. *P* values were calculated by using two-sided paired t tests, one-way ANOVA (followed by Tukey’s post-hoc test), repeated measures (RM) one-way ANOVA (followed by Dunnet post-hoc test), a mixed model for RM one-way ANOVA, two-way ANOVA followed by Šidák’s multiple comparison or Friedman test (followed by Dunns post-hoc test). Statistical analysis was performed using Graphpad PRISM version 9.1.0 with significance set at *P < 0.05, **P < 0.01; ***P < 0.001. All statistical information, such as tests, the value of n, and precision measures (mean ± SD) are specified in the figure legends. Because of biological variation between primary material samples, different numbers of patients and healthy donors were measured between experiments. Unequal replicate numbers introduce a systematic statistical bias, thereby penalizing significance testing in conditions with lower number of replicates even when the biological effect is stronger.

### Ethics approval and Consent to participate

After obtaining written informed consent, patient blood was collected during diagnostic or follow-up procedures at the Department of Hematology of the Academic Medical Center Amsterdam. This study was approved by the AMC Ethical Review Board under the number METC 2013/159 and conducted in accordance with the Declaration of Helsinki. Patient confidentiality was maintained by anonymization of all patient samples by our biobank to prevent identification.

## Supplementary information

Supplementary figures

Supplementary Figure file 2

Supplementary table 1

Supplementary table 2

Supplementary table 3

## Data Availability

All analyses and data generated during this study are included in this article and its Supplementary Information files. Full-length, uncropped original western blot images are provided in Supplementary Information as well. Further information is available from the corresponding author upon reasonable request.

## References

[1] Bose P, Gandhi V, Konopleva M. Pathways and mechanisms of venetoclax resistance. Leuk Lymphoma. 2017;58:1–17. 28140720 10.1080/10428194.2017.1283032 PMC5478500

[2] Furman RR, Cheng S, Lu P, Setty M, Perez AR, Guo A, et al. Ibrutinib resistance in chronic lymphocytic leukemia. N Engl J Med. 2014;370:2352–4. 24869597 10.1056/NEJMc1402716 PMC4512173

[3] van Bruggen JAC, Martens AWJ, Tonino SH, Kater AP. Overcoming the hurdles of autologous T-cell-based therapies in B-cell non-hodgkin lymphoma. Cancers. 2020;12:3837. 10.3390/cancers12123837 PMC7765898 33353234

[4] Thijssen R, Slinger E, Weller K, Geest CR, Beaumont T, van Oers MH, et al. Resistance to ABT-199 induced by microenvironmental signals in chronic lymphocytic leukemia can be counteracted by CD20 antibodies or kinase inhibitors. Haematologica. 2015;100:e302–6. 25957396 10.3324/haematol.2015.124560 PMC5004430

[5] Tsuyama N, Sakata S, Baba S, Mishima Y, Nishimura N, Ueda K, et al. BCL2 expression in DLBCL: reappraisal of immunohistochemistry with new criteria for therapeutic biomarker evaluation. Blood. 2017;130:489–500. 28522442 10.1182/blood-2016-12-759621

[6] Del Gaizo Moore V, Brown JR, Certo M, Love TM, Novina CD, Letai A. Chronic lymphocytic leukemia requires BCL2 to sequester prodeath BIM, explaining sensitivity to BCL2 antagonist ABT-737. J Clin Investig. 2007;117:112–21. 17200714 10.1172/JCI28281 PMC1716201

[7] Kitada S, Andersen J, Akar S, Zapata JM, Takayama S, Krajewski S, et al. Expression of apoptosis-regulating proteins in chronic lymphocytic leukemia: correlations with In vitro and In vivo chemoresponses. Blood. 1998;91:3379–89. 9558396

[8] Tsujimoto Y, Cossman J, Jaffe E, Croce CM. Involvement of the bcl-2 gene in human follicular lymphoma. Science. 1985;228:1440–3. 3874430 10.1126/science.3874430

[9] Hofmann WK, de Vos S, Tsukasaki K, Wachsman W, Pinkus GS, Said JW, et al. Altered apoptosis pathways in mantle cell lymphoma detected by oligonucleotide microarray. Blood. 2001;98:787–94. 11468180 10.1182/blood.v98.3.787

[10] Berndt SI, Skibola CF, Joseph V, Camp NJ, Nieters A, Wang Z, et al. Genome-wide association study identifies multiple risk loci for chronic lymphocytic leukemia. Nat Genet. 2013;45:868–76. 23770605 10.1038/ng.2652 PMC3729927

[11] Shin MS, Kim HS, Kang CS, Park WS, Kim SY, Lee SN, et al. Inactivating mutations of CASP10 gene in non-Hodgkin lymphomas. Blood. 2002;99:4094–9. 12010812 10.1182/blood.v99.11.4094

[12] Martens AWJ, Janssen SR, Derks IAM, Adams Iii HC, Izhak L, van Kampen R, et al. CD3xCD19 DART molecule treatment induces non-apoptotic killing and is efficient against high-risk chemotherapy and venetoclax-resistant chronic lymphocytic leukemia cells. J Immunother Cancer. 2020;8:e000218. 10.1136/jitc-2019-000218 PMC7319711 32581054

[13] Trapani JA, Smyth MJ. Functional significance of the perforin/granzyme cell death pathway. Nat Rev Immunol. 2002;2:735–47. 12360212 10.1038/nri911

[14] Kagi D, Vignaux F, Ledermann B, Burki K, Depraetere V, Nagata S, et al. Fas and perforin pathways as major mechanisms of T cell-mediated cytotoxicity. Science. 1994;265:528–30. 7518614 10.1126/science.7518614

[15] Cullen SP, Martin SJ. Mechanisms of granule-dependent killing. Cell Death Differ. 2008;15:251–62. 17975553 10.1038/sj.cdd.4402244

[16] Cullen SP, Brunet M, Martin SJ. Granzymes in cancer and immunity. Cell Death Differ. 2010;17:616–23. 20075940 10.1038/cdd.2009.206

[17] Heusel JW, Wesselschmidt RL, Shresta S, Russell JH, Ley TJ. Cytotoxic lymphocytes require granzyme B for the rapid induction of DNA fragmentation and apoptosis in allogeneic target cells. Cell. 1994;76:977–87. 8137431 10.1016/0092-8674(94)90376-x

[18] Waterhouse NJ, Sedelies KA, Browne KA, Wowk ME, Newbold A, Sutton VR, et al. A central role for Bid in granzyme B-induced apoptosis. J Biol Chem. 2005;280:4476–82. 15574417 10.1074/jbc.M410985200

[19] Darmon AJ, Nicholson DW, Bleackley RC. Activation of the apoptotic protease CPP32 by cytotoxic T-cell-derived granzyme B. Nature. 1995;377:446–8. 7566124 10.1038/377446a0

[20] Afonina IS, Cullen SP, Martin SJ. Cytotoxic and non-cytotoxic roles of the CTL/NK protease granzyme B. Immunol Rev. 2010;235:105–16. 20536558 10.1111/j.0105-2896.2010.00908.x

[21] Hassin D, Garber OG, Meiraz A, Schiffenbauer YS, Berke G. Cytotoxic T lymphocyte perforin and Fas ligand working in concert even when Fas ligand lytic action is still not detectable. Immunology. 2011;133:190–6. 21517838 10.1111/j.1365-2567.2011.03426.x PMC3088981

[22] Miao EA, Rajan JV, Aderem A. Caspase-1-induced pyroptotic cell death. Immunol Rev. 2011;243:206–14. 21884178 10.1111/j.1600-065X.2011.01044.x PMC3609431

[23] Zhou Z, He H, Wang K, Shi X, Wang Y, Su Y, et al. Granzyme A from cytotoxic lymphocytes cleaves GSDMB to trigger pyroptosis in target cells. Science. 2020;368:eaaz7548. 10.1126/science.aaz7548 32299851

[24] Snyder AG, Hubbard NW, Messmer MN, Kofman SB, Hagan CE, Orozco SL, et al. Intratumoral activation of the necroptotic pathway components RIPK1 and RIPK3 potentiates antitumor immunity. Sci Immunol. 2019;4:eaaw2004. 10.1126/sciimmunol.aaw2004 PMC6831211 31227597

[25] Kearney CJ, Martin SJ. An inflammatory perspective on necroptosis. Mol Cell. 2017;65:965–73. 28306512 10.1016/j.molcel.2017.02.024

[26] Yatim N, Jusforgues-Saklani H, Orozco S, Schulz O, Barreira da Silva R, Reis e Sousa C, et al. RIPK1 and NF-kappaB signaling in dying cells determines cross-priming of CD8(+) T cells. Science. 2015;350:328–34. 26405229 10.1126/science.aad0395 PMC4651449

[27] Aaes TL, Kaczmarek A, Delvaeye T, De Craene B, De Koker S, Heyndrickx L, et al. Vaccination with necroptotic cancer cells induces efficient anti-tumor immunity. Cell Rep. 2016;15:274–87. 27050509 10.1016/j.celrep.2016.03.037

[28] Favaro F, Both D, Derks IAM, Spaargaren M, Munoz-Pinedo C, Eldering E. Negligible role of TRAIL death receptors in cell death upon endoplasmic reticulum stress in B-cell malignancies. Oncogenesis. 2023;12:6. 36755015 10.1038/s41389-023-00450-w PMC9908905

[29] Kataoka T, Shinohara N, Takayama H, Takaku K, Kondo S, Yonehara S, et al. Concanamycin A, a powerful tool for characterization and estimation of contribution of perforin- and Fas-based lytic pathways in cell-mediated cytotoxicity. J Immunol. 1996;156:3678–86. 8621902

[30] Walsh RM, Ambrose J, Jack JL, Eades AE, Bye BA, Tannus Ruckert M, et al. Depletion of tumor-derived CXCL5 improves T cell infiltration and anti-PD-1 therapy response in an obese model of pancreatic cancer. J Immunother Cancer. 2025;13:e010057. 10.1136/jitc-2024-010057 PMC11931939 40121029

[31] Alam MS, Otsuka S, Wong N, Abbasi A, Gaida MM, Fan Y, et al. TNF plays a crucial role in inflammation by signaling via T cell TNFR2. Proc Natl Acad Sci USA. 2021;118:e2109972118. 10.1073/pnas.2109972118 PMC8685675 34873037

[32] Kroemer G, Galluzzi L, Brenner C. Mitochondrial membrane permeabilization in cell death. Physiol Rev. 2007;87:99–163. 17237344 10.1152/physrev.00013.2006

[33] Fritsch M, Gunther SD, Schwarzer R, Albert MC, Schorn F, Werthenbach JP, et al. Caspase-8 is the molecular switch for apoptosis, necroptosis and pyroptosis. Nature. 2019;575:683–7. 31748744 10.1038/s41586-019-1770-6

[34] Han T, Ruan C, Lin H, Zhang Y, Li L, Sun YH, et al. RIPK1 S161 phosphorylation promotes further autophosphorylation and cecal necroptosis in TNF-treated mice. J Exp Med. 2025;222:e20250277. 10.1084/jem.20250277 40996440

[35] Koerner L, Li X, Silnov E, Laurien L, Pasparakis M. RIPK1 autophosphorylation at S161 mediates cell death and inflammation. J Exp Med. 2025;222:e20250277. 10.1084/jem.20250279 PMC12462663 40996439

[36] Degterev A, Huang Z, Boyce M, Li Y, Jagtap P, Mizushima N, et al. Chemical inhibitor of nonapoptotic cell death with therapeutic potential for ischemic brain injury. Nat Chem Biol. 2005;1:112–9. 16408008 10.1038/nchembio711

[37] Mandal P, Berger SB, Pillay S, Moriwaki K, Huang C, Guo H, et al. RIP3 induces apoptosis independent of pronecrotic kinase activity. Mol Cell. 2014;56:481–95. 25459880 10.1016/j.molcel.2014.10.021 PMC4512186

[38] Sun L, Wang H, Wang Z, He S, Chen S, Liao D, et al. Mixed lineage kinase domain-like protein mediates necrosis signaling downstream of RIP3 kinase. Cell. 2012;148:213–27. 22265413 10.1016/j.cell.2011.11.031

[39] Newton K, Wickliffe KE, Dugger DL, Maltzman A, Roose-Girma M, Dohse M, et al. Cleavage of RIPK1 by caspase-8 is crucial for limiting apoptosis and necroptosis. Nature. 2019;574:428–31. 31511692 10.1038/s41586-019-1548-x

[40] Kaczmarek A, Vandenabeele P, Krysko DV. Necroptosis: the release of damage-associated molecular patterns and its physiological relevance. Immunity. 2013;38:209–23. 23438821 10.1016/j.immuni.2013.02.003

[41] Kimman T, Cuenca M, Tieland RG, Rockx-Brouwer D, Janssen J, Motais B, et al. Engineering anti-BCMA CAR T cells for enhancing myeloma killing efficacy via apoptosis regulation. Nat Commun. 2025;16:4638. 40389394 10.1038/s41467-025-59818-8 PMC12089368

[42] Medema JP, Toes RE, Scaffidi C, Zheng TS, Flavell RA, Melief CJ, et al. Cleavage of FLICE (caspase-8) by granzyme B during cytotoxic T lymphocyte-induced apoptosis. Eur J Immunol. 1997;27:3492–8. 9464839 10.1002/eji.1830271250

[43] Grootjans S, Vanden Berghe T, Vandenabeele P. Initiation and execution mechanisms of necroptosis: an overview. Cell Death Differ. 2017;24:1184–95. 28498367 10.1038/cdd.2017.65 PMC5520172

[44] Yao K, Shi Z, Zhao F, Tan C, Zhang Y, Fan H, et al. RIPK1 in necroptosis and recent progress in related pharmaceutics. Front Immunol. 2025;16:1480027. 40007541 10.3389/fimmu.2025.1480027 PMC11850271

[45] Dufva O, Koski J, Maliniemi P, Ianevski A, Klievink J, Leitner J, et al. Integrated drug profiling and CRISPR screening identify essential pathways for CAR T-cell cytotoxicity. Blood. 2020;135:597–609. 31830245 10.1182/blood.2019002121 PMC7098811

[46] Zhang Z, Kong X, Ligtenberg MA, van Hal-van Veen SE, Visser NL, de Bruijn B, et al. RNF31 inhibition sensitizes tumors to bystander killing by innate and adaptive immune cells. Cell Rep Med. 2022;3:100655. 35688159 10.1016/j.xcrm.2022.100655 PMC9245005

[47] Chun N, Ang RL, Chan M, Fairchild RL, Baldwin WM, 3rd, et al. T cell-derived tumor necrosis factor induces cytotoxicity by activating RIPK1-dependent target cell death. JCI Insight. 2021;6:e148643. 10.1172/jci.insight.148643 PMC8783689 34752416

[48] Boulch M, Cazaux M, Cuffel A, Guerin MV, Garcia Z, Alonso R, et al. Tumor-intrinsic sensitivity to the pro-apoptotic effects of IFN-gamma is a major determinant of CD4(+) CAR T-cell antitumor activity. Nat Cancer. 2023;4:968–83. 37248395 10.1038/s43018-023-00570-7 PMC10368531

[49] Padhan K, Varma R. Immunological synapse: a multi-protein signalling cellular apparatus for controlling gene expression. Immunology. 2010;129:322–8. 20409153 10.1111/j.1365-2567.2009.03241.x PMC2826677

[50] Hombach A, Kohler H, Rappl G, Abken H. Human CD4+ T cells lyse target cells via granzyme/perforin upon circumvention of MHC class II restriction by an antibody-like immunoreceptor. J Immunol. 2006;177:5668–75. 17015756 10.4049/jimmunol.177.8.5668

[51] Degterev A, Hitomi J, Germscheid M, Ch’en IL, Korkina O, Teng X, et al. Identification of RIP1 kinase as a specific cellular target of necrostatins. Nat Chem Biol. 2008;4:313–21. 18408713 10.1038/nchembio.83 PMC5434866

[52] He S, Wang L, Miao L, Wang T, Du F, Zhao L, et al. Receptor interacting protein kinase-3 determines cellular necrotic response to TNF-alpha. Cell. 2009;137:1100–11. 19524512 10.1016/j.cell.2009.05.021

[53] Dillon CP, Weinlich R, Rodriguez DA, Cripps JG, Quarato G, Gurung P, et al. RIPK1 blocks early postnatal lethality mediated by caspase-8 and RIPK3. Cell. 2014;157:1189–202. 24813850 10.1016/j.cell.2014.04.018 PMC4068710

[54] Tait SW, Oberst A, Quarato G, Milasta S, Haller M, Wang R, et al. Widespread mitochondrial depletion via mitophagy does not compromise necroptosis. Cell Rep. 2013;5:878–85. 24268776 10.1016/j.celrep.2013.10.034 PMC4005921

[55] Moujalled DM, Cook WD, Okamoto T, Murphy J, Lawlor KE, Vince JE, et al. TNF can activate RIPK3 and cause programmed necrosis in the absence of RIPK1. Cell Death Dis. 2013;4:e465. 23328672 10.1038/cddis.2012.201 PMC3563989

[56] Cho YS, Challa S, Moquin D, Genga R, Ray TD, Guildford M, et al. Phosphorylation-driven assembly of the RIP1-RIP3 complex regulates programmed necrosis and virus-induced inflammation. Cell. 2009;137:1112–23. 19524513 10.1016/j.cell.2009.05.037 PMC2727676

[57] Yang Y, Wu M, Cao D, Yang C, Jin J, Wu L, et al. ZBP1-MLKL necroptotic signaling potentiates radiation-induced antitumor immunity via intratumoral STING pathway activation. Sci Adv. 2021;7:eabf6290. 34613770 10.1126/sciadv.abf6290 PMC8494295

[58] Zhang T, Yin C, Fedorov A, Qiao L, Bao H, Beknazarov N, et al. ADAR1 masks the cancer immunotherapeutic promise of ZBP1-driven necroptosis. Nature. 2022;606:594–602. 35614224 10.1038/s41586-022-04753-7 PMC9373927

[59] Song Q, Fan Y, Zhang H, Wang N. Z-DNA binding protein 1 orchestrates innate immunity and inflammatory cell death. Cytokine Growth Factor Rev. 2024;77:15–29. 38548490 10.1016/j.cytogfr.2024.03.005

[60] Crawford MP, Sinha S, Renavikar PS, Borcherding N, Karandikar NJ. CD4 T cell-intrinsic role for the T helper 17 signature cytokine IL-17: Effector resistance to immune suppression. Proc Natl Acad Sci USA. 2020;117:19408–14. 32719138 10.1073/pnas.2005010117 PMC7430972

[61] de Jong AJ, Pollastro S, Kwekkeboom JC, Andersen SN, Dorjee AL, Bakker AM, et al. Functional and phenotypical analysis of IL-6-secreting CD4(+) T cells in human adipose tissue. Eur J Immunol. 2018;48:471–81. 29283192 10.1002/eji.201747037 PMC5873429

[62] Dicharry D, Malek AE. Unlocking the role of Treg cells immune response and infectious risk following CAR T-cell therapy in patients with cancer. Int J Mol Sci. 2025;26:1602. 10.3390/ijms26041602 PMC11854923 40004068

[63] Couper KN, Blount DG, Riley EM. IL-10: the master regulator of immunity to infection. J Immunol. 2008;180:5771–7. 18424693 10.4049/jimmunol.180.9.5771

[64] Siddiqi T, Soumerai JD, Dorritie KA, Stephens DM, Riedell PA, Arnason JE, et al. Updated follow-up of patients with relapsed/refractory chronic lymphocytic leukemia/small lymphocytic lymphoma treated with lisocabtagene maraleucel in the phase 1 monotherapy cohort of transcend CLL 004, including high-risk and ibrutinib-treated patients. Blood. 2020;136:40–1.

[65] Chen DJ, Huerta S. Smac mimetics as new cancer therapeutics. Anticancer Drugs. 2009;20:646–58. 19550293 10.1097/CAD.0b013e32832ced78

[66] Michie J, Kearney CJ, Hawkins ED, Silke J, Oliaro J. The immuno-modulatory effects of inhibitor of apoptosis protein antagonists in cancer immunotherapy. Cells. 2020;9:207. 10.3390/cells9010207 PMC7017284 31947615

[67] Michie J, Beavis PA, Freeman AJ, Vervoort SJ, Ramsbottom KM, Narasimhan V, et al. Antagonism of IAPs enhances CAR T-cell Efficacy. Cancer Immunol Res. 2019;7:183–92. 30651288 10.1158/2326-6066.CIR-18-0428

[68] McComb S, Aguade-Gorgorio J, Harder L, Marovca B, Cario G, Eckert C, et al. Activation of concurrent apoptosis and necroptosis by SMAC mimetics for the treatment of refractory and relapsed ALL. Sci Transl Med. 2016;8:339ra70. 27194728 10.1126/scitranslmed.aad2986

[69] O’Neill KL, Huang K, Zhang J, Chen Y, Luo X. Inactivation of prosurvival Bcl-2 proteins activates Bax/Bak through the outer mitochondrial membrane. Genes Dev. 2016;30:973–88. 27056669 10.1101/gad.276725.115 PMC4840302

[70] Horn S, Hughes MA, Schilling R, Sticht C, Tenev T, Ploesser M, et al. Caspase-10 negatively regulates caspase-8-mediated cell death, switching the response to CD95L in favor of NF-kappaB activation and cell survival. Cell Rep. 2017;19:785–97. 28445729 10.1016/j.celrep.2017.04.010 PMC5413585

[71] Hart T, Chandrashekhar M, Aregger M, Steinhart Z, Brown KR, MacLeod G, et al. High-resolution CRISPR screens reveal fitness genes and genotype-specific cancer liabilities. Cell. 2015;163:1515–26. 26627737 10.1016/j.cell.2015.11.015

[72] Katsarou A, Sjostrand M, Naik J, Mansilla-Soto J, Kefala D, Kladis G, et al. Combining a CAR and a chimeric costimulatory receptor enhances T cell sensitivity to low antigen density and promotes persistence. Sci Transl Med. 2021;13:eabh1962. 34878825 10.1126/scitranslmed.abh1962 PMC9869449

[73] van der Schans JJ, Katsarou A, Kladis G, Bar C, Ramirez MM, Themeli M, et al. A convenient viral transduction based method for advanced multi-engineering of primary human (CAR) T-cells. J Genet Eng Biotechnol. 2024;22:100446. 39674638 10.1016/j.jgeb.2024.100446 PMC11629549

