# Supplementary material for: Necroptotic cell death and immunomodulator release induced by T-cell engaging anti-lymphoma therapies
Source: Cell Death Dis. 2026 Jun 25;17(1):786. doi: 10.1038/s41419-026-08993-7 (PMC13558721; doi:10.1038/s41419-026-08993-7)
Supplement: Supplementary file 1 [file 41419_2026_8993_MOESM3_ESM.pdf]

ID	Sex	Age	Rai stage	Mutation status	ALC (×109/L)	%CD5+CD19+	Cytogenetics	TP53 mutation	Treatment
1	M	60	0	Unmutated	90.91	96.16	NA	WT	None
2	M	81	I	Mutated	149.4	98.2	del(11q22), del (13q4)	WT	None
3	F	79	0	Mutated	85.81	95.92	del (13q4)	NA	None
