# Supplementary material for: Necroptotic cell death and immunomodulator release induced by T-cell engaging anti-lymphoma therapies
Source: Cell Death Dis. 2026 Jun 25;17(1):786. doi: 10.1038/s41419-026-08993-7 (PMC13558721; doi:10.1038/s41419-026-08993-7)
Supplement: Supplementary file 2 [file 41419_2026_8993_MOESM4_ESM.pdf]

Gene name	Target sequence	Exon	Reference
BAK1	ACGGCAGCTCGCCATCATCG	5	O'Neill, 2016 #64
BAX	CTGCAGGATGATTGCCGCCG	4	O'Neill, 2016 #64
BID	GCTCATCGTAGCCCTCCCAC	3	O'Neill, 2016 #64
CASP8	GCCTGGACTACATTCCGCAA	2	Horn, 2017 #64
RIPK1	GATGCACGTGCTGAAAGCCG	3	Hart, 2015 #65
	AATGCAGTGCACCAGCCCTC	9	Hart, 2015 #65
	GGAAGGCATAAAGGGAGCCA	11	Hart, 2015 #65
RIPK3	AACCAGGAGCTCGTCGGCAA	2	Hart, 2015 #65
	TAGGAAGGCGATATCCAGGG	3	Hart, 2015 #65
	GGCCTGTCCACATTTCAGGG	4	Hart, 2015 #65
MLKL	TTTGTGGATGACCTGGCCAA	2	Hart, 2015 #65
	GCAAATCAAGGAGATCAAGA	4	Hart, 2015 #65
	AGTGAGGCAGACTTTCAATA	5	Hart, 2015 #65
