# Supplementary material for: Necroptotic cell death and immunomodulator release induced by T-cell engaging anti-lymphoma therapies
Source: Cell Death Dis. 2026 Jun 25;17(1):786. doi: 10.1038/s41419-026-08993-7 (PMC13558721; doi:10.1038/s41419-026-08993-7)
Supplement: Supplementary file 3 [file 41419_2026_8993_MOESM5_ESM.pdf]

Target	Supplier	Cat number	Technique
Bax	Cell Signalling	5023	Western Blot
Bak	Cell Signalling	3814S	Western Blot
Bid	Cell Signalling	2002	Western Blot
RIPK1	Cell Signalling	3493	Western Blot
RIPK3	Cell Signalling	13526	Western Blot
MLKL	Abcam	Ab184718	Western Blot
Caspase-8	Santa Cruz Biotechnolog	sc-6136	Western Blot
Gasdermin B	Abcam	ab215729	Western Blot
Gasdermin D	Abcam	ab210070	Western Blot
β-actin	Santa Cruz Biotechnolog	664802	Western Blot
IRDye 800 CW donkey anti-mouse IgG (H + L)	Li-Cor	926-32212	Western Blot
IRDye 800 CW donkey anti-goat IgG (H + L)	Li-Cor	926-32214	Western Blot
IRDye680LT donkey anti-rabbit	Li-Cor	926-68023	Western Blot
CD3	eBioscience	56-0038-82	Flow Cytometry
CD4	Biolegend	300536	Flow Cytometry
CD8	eBioscience	25-0088-42	Flow Cytometry
IL-6	Biolegend	MQ2-13A5/501123	Flow Cytometry
IL-10	Biolegend	JES3-9D7	Flow Cytometry
IL-17	Biolegend	BL168/512322	Flow Cytometry
CD95	BD Pharmingen	555673	Flow Cytometry
Phospho-RIPK1 (Ser161)	Proteintech	CL488-66854	Flow Cytometry
